# Using the Cardiac–Electrophysiological Balance Index to Predict Arrhythmia Risk After Colonoscopy

**DOI:** 10.3390/medicina61010013

**Published:** 2024-12-26

**Authors:** Seyit Ali Volkan Polatkan, Seyda Gunay-Polatkan, Ozgen Isik, Deniz Sigirli

**Affiliations:** 1 Department of General Surgery, Faculty of Medicine, Bursa Uludag University, 16059 Bursa, Turkey; ozgenisik@uludag.edu.tr; 2 Department of Cardiology, Faculty of Medicine, Bursa Uludag University, 16059 Bursa, Turkey; seydagunay@uludag.edu.tr; 3 Department of Biostatistics, Faculty of Medicine, Bursa Uludag University, 16059 Bursa, Turkey; sigirli@uludag.edu.tr

**Keywords:** colonoscopy, bowel preparation, arrhythmia, heart, electrolyte

## Abstract

*Background and Objectives:* Colorectal cancer is the second leading cause of cancer-related deaths in the U.S., and colonoscopy is a critical tool for colon cancer screening and diagnosis. Electrolyte disturbances and autonomic nervous system dysfunction that may occur due to bowel preparation and the colonoscopy procedure itself may play a role in the development of cardiac arrhythmia. This study aimed to assess the index of cardiac–electrophysiological balance (iCEB) to predict ventricular arrhythmia risk related to colonoscopy. *Materials and Methods:* Patients undergoing elective colonoscopy with a normal sinus rhythm were included. Electrocardiography (ECG) recordings both before bowel preparation and after the colonoscopy procedure were obtained. Values of the index of cardiac–electrophysiological balance (iCEB) were compared. *Results:* Among 36 patients, it was determined that the heart rate values of the patients before bowel preparation were higher than the heart rate values after colonoscopy [74.5 (60–108) bpm vs. 68.5 (53–108) bpm, *p* = 0.021]. The duration of QT interval increased (370.9 ± 27.8 ms vs. 398.7 ± 29.4 ms, *p* < 0.001) and the iCEB increased from 4.1 ± 0.5 to 4.5 ± 0.6 (*p* < 0.001), indicating a significant post-procedural risk of ventricular arrhythmias. *Conclusions:* These findings suggest that routine iCEB assessment post-colonoscopy could identify high-risk patients requiring closer monitoring.

## 1. Introduction

Colorectal cancer is the second leading cause of cancer-related deaths in the U.S. [1]. Colonoscopy is a critical tool for colon cancer screening and diagnosis globally, with over 15 million procedures conducted annually in the U.S. [2]. The effectiveness of colonoscopy is largely determined by the quality of colonic mucosa visualization [3]. Residual fecal matter can obstruct the view of colorectal lesions, highlighting the necessity of adequate bowel preparation. Various bowel preparation agents have been developed to improve imaging, including high-volume and low-volume polyethylene glycol (PEG) and sulfate-free PEG–electrolyte solutions [4,5].

Despite their general tolerance, bowel preparation agents can lead to adverse effects such as electrolyte imbalances and, less commonly, serious cardiac events like heart failure exacerbations, arrhythmias, and cardiac arrest [6,7,8,9,10]. Additional neuronal factors aside from electrolyte disturbances, including elevated sympathetic and parasympathetic activity, are also present, and the simultaneous activation of both sympathetic and parasympathetic systems is a known trigger for cardiac arrhythmia [11,12,13,14,15].

Cardiac ECG changes related to colonoscopy are typically benign and transient, such as ST-segment and T-wave changes [16]. However, severe cases, including polymorphic ventricular tachycardia and ventricular fibrillation related with colonoscopy, have been reported [17,18]. Although such severe arrhythmias are rare and usually occur in high-risk patients with comorbidities or significant electrolyte disturbances [17,18,19], Kajy et al. documented new-onset supraventricular tachycardia in patients with normal electrolyte panels and without a history of cardiac arrhythmia [20].

Even though electrolyte disturbances due to bowel preparation are often asymptomatic, individuals with pre-existing cardiac or renal conditions are at higher risk for arrhythmias due to an electrolyte imbalance. Ho JM et al. observed an increased risk of hypokalemia following bowel preparation in older patients [21]. Additionally, excessive fluid intake with low dietary solutes can lead to hyponatremia [22,23]. There are a few research studies which have looked at how serum electrolyte levels differ before and after bowel preparation [24,25]. As the current guidelines do not include recommendations for pre- or post-colonoscopy electrolyte measurement [26], undetected but present electrolyte abnormalities may trigger arrhythmias both in hospital and even after discharge [27].

It is well known that most research excludes older adults and patients with renal or cardiac disease, and this causes real-life risks in these high-risk groups to be overlooked [28,29]. As the number of colonoscopies for screening and surveillance increase, the number of high-risk patients undergoing colonoscopy procedures will also increase. Some patients who are discharged on the same day after a colonoscopy procedure may be discharged with unnoticed health conditions which may trigger cardiac arrhythmia. Because of this, simple, non-invasive, and easily accessible electrocardiographic parameters could be valuable for identifying patients at risk of arrhythmias before discharge. The QT interval, which reflects ventricular depolarization and repolarization, is a well-known ECG parameter that can be used to assess the risk of drug-induced arrhythmias [30]. The index of cardiac–electrophysiological balance (iCEB) is a relatively new and more sensitive risk marker for malignant arrhythmias. The iCEB focuses on the balance between contraction and relaxation, whereas the QT interval represents a single cycle of ventricular contraction and relaxation. Researchers have reported that the iCEBc was altered more significantly and earlier than the patient’s corresponding change in QTc [31,32].

This study aimed to evaluate the index of cardiac–electrophysiological balance (iCEB) to evaluate arrhythmia risk in patients undergoing elective colonoscopy before discharge.

## 2. Material and Method

### 2.1. Study Design

In this prospective, observational, single-center study, we included adult patients undergoing elective colonoscopy for cancer screening or follow-up between 25 September 2023 and 1 March 2024. All participants had a normal sinus rhythm. We excluded individuals with atrial fibrillation, pre-excitation findings, bundle branch blocks, atrioventricular blocks, or a pacemaker rhythm. Patients who were on QT-prolonging medications or had a history of cardiac arrhythmias or heart failure were also excluded. We documented clinical characteristics, recorded ECGs before bowel preparation and after colonoscopy, noted comorbidities, and collected laboratory data. This study was conducted in accordance with the principles of the Declaration of Helsinki, and the protocol was approved by the Institutional Ethics Committee of Bursa Uludag University (approval number 2023-17/41, dated 19 September 2023). All data were fully anonymized before access and a signed informed written consent form was obtained from all participants.

### 2.2. ECG Examination

Each participant underwent a 12-lead ECG using a GE Healthcare MAC 200 Resting ECG System (GE Healthcare, Milwaukee, WI, USA) both before bowel preparation and after the colonoscopy procedure. The ECG was recorded at a paper speed of 50 mm/s. The scanned ECG images were loaded into a computer and analyzed using Adobe Photoshop CS6 software (Adobe Systems, Inc., San Jose, CA, USA). Two cardiologists independently reviewed the ECGs manually. Measurements were taken from lead II and lead V5. Key ECG parameters including the QRS interval, QT interval, corrected QT (QTc) interval, iCEB, and corrected iCEB (iCEBc) were calculated. The QRS interval was measured from the end of the PR interval to the end of the S-wave. R–R intervals were determined by measuring the time between consecutive R peaks. The QT interval was calculated from the start of the QRS complex to the end of the T wave [33] and corrected for heart rate using the Bazett formula (QTcB = QT/√RR) [34]. iCEB was computed by dividing the QT interval by the QRS duration (QT/QRS), and iCEBc was derived by dividing the QTc interval by the QRS duration [QTc/QRS] [31,32].

### 2.3. Statistical Analysis

The normality of data was assessed using the Shapiro–Wilk test. Data that followed a normal distribution were expressed as the mean ± standard deviation. Paired sample *t*-tests were used for comparisons between two dependent groups for normally distributed variables. Data that were not normally distributed were presented as the median (Q1–Q3) and analyzed using the Wilcoxon test for paired samples. Categorical data were described with counts and percentages. The significance level was set at α = 0.05. All statistical analyses were conducted using IBM SPSS Statistics version 23.0.

## 3. Results

Among the 36 patients who underwent elective colonoscopy, 16 patients (44.4%) were female, and the mean (SD) age was 55.1 ± 11.2 years. Serum electrolyte levels of all patients were in a normal range. The clinical and laboratory findings are shown in Table 1.

**Figure 1:**
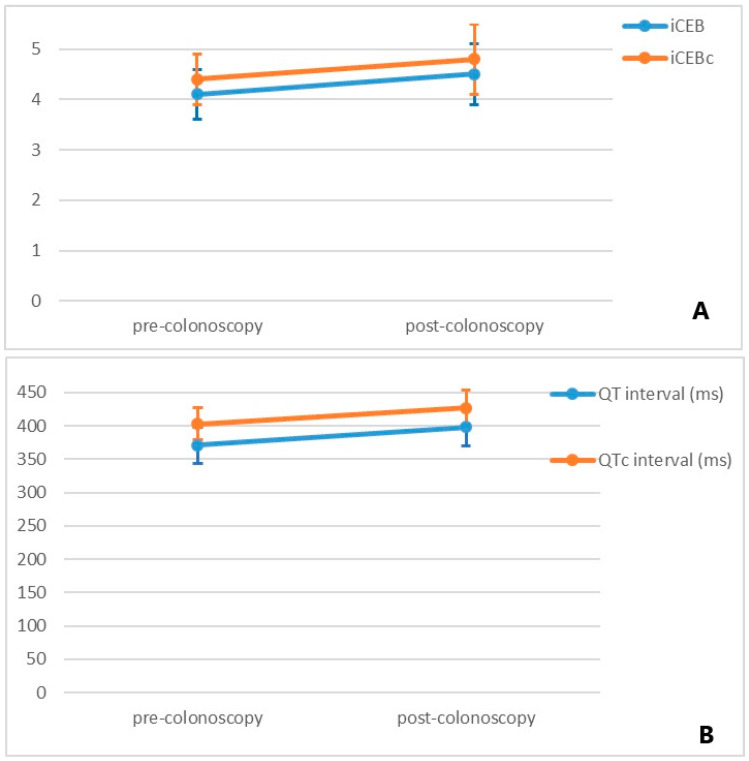
Change in ventricular repolarization parameters after colonoscopy ((**A**) for iCEB and iCEBc, (**B**) for QT and QTc intervals). Points and error bars represent mean ± 1 standard deviation.

It was determined that the heart rate values of the patients before bowel preparation were higher than the heart rate values after colonoscopy (median (minimum–maximum), 74.5 (60–108) bpm vs. 68.5 (53–108) bpm, *p* = 0.021). While QRS durations were similar (median (minimum–maximum), 91.0 (73–120) ms vs. 90.5 (74–132) ms, *p* = 0.317), the increase in the duration of QT interval was statistically significant (mean ± [SD], 370.9 ± 27.8 ms vs. 398.7 ± 29.4 ms, *p* < 0.001). Also, the values of both iCEB (mean ± [SD], 4.1 ± 0.5 vs. 4.5 ± 0.6, *p* < 0.001) and iCEBc (mean ± [SD], 4.4 ± 0.5 vs. 4.8 ± 0.7, *p* <0.001) were higher in the post-colonoscopy group. The ECG parameters of the study population are listed in Table 2 and Figure 1.

## 4. Discussion

Our study revealed that, alongside the QT and QTc intervals, both iCEB and iCEBc statistically significantly increased after the colonoscopy procedure. In the literature, there is previous research reporting an increased iCEB related to cardiac arrhythmia risk in patients with various medical conditions, such as type 2 diabetes, coronary artery disease, renal failure, and COVID-19 [35,36,37,38]. To the best of our knowledge, our study is the first to investigate the change in iCEB in patients undergoing elective colonoscopy.

Three fundamental mechanisms underlie the pathophysiology of cardiac arrhythmias: (1) increased or decreased automaticity, (2) triggered activity, or (3) re-entry. Automaticity can be suppressed or enhanced by a number of factors, such as heart ischemia, heart failure, electrolyte imbalance, dehydration, medications, aging, and anxiety. Multiple arrhythmias for both atrial and ventricular arrhythmias may arise from increased automaticity. The triggered activity, which typically follows earlier and delayed depolarizations, causes repeated spontaneous depolarizations and precipitates ventricular arrhythmias [27].

Colonoscopy remains the preferred method for evaluating the colon, and the procedure may be associated with the development of cardiac arrhythmias because bowel preparation prior to colonoscopy may trigger dehydration and an electrolyte imbalance. In addition, the colonoscopy procedure affects the autonomic nervous system. Procedural anxiety triggers sympathetic activity, while parasympathetic activity is also increased due to increased gastrointestinal motility and increased levels of colonic secretions [11,12,13,14]. Furthermore, some patients undergoing colonoscopy are at risk for arrhythmias because they are older and have comorbidities, as exemplified above.

Electrolyte imbalances associated with colonoscopy may include hyponatremia, hypocalcemia, hypokalemia, and hypomagnesemia [21,39,40]. While most electrolyte disturbances due to bowel preparation are asymptomatic, symptomatic and even fatal cases have been documented [41,42,43,44,45]. Reumkens A. et al. reported that 23.6% of “high-risk” patients experienced hypokalemia following bowel preparation, with two cases of severe post-colonoscopy hypokalemia leading to fatal ventricular arrhythmias [44,46]. Mild hypokalemia is often asymptomatic, but more severe cases can be symptomatic, including constipation, fatigue, and life-threatening ventricular arrhythmias [47,48,49]. The risk profiles for patients developing hypokalemia after bowel preparation are not well established, though elderly and dehydrated patients may be more susceptible [50]. Patients with heart failure, cardiac ischemia, or a history of arrhythmias are known to be at a higher risk of fatal arrhythmias when they are hypokalemic [49]. Physicians who perform colonoscopy should be aware of high-risk health conditions and drugs affecting serum electrolyte levels (diuretics, angiotensin-converting enzyme inhibitors, angiotensin II receptor blockers, chemotherapy drugs, antibiotics, etc.). Even though current clinical guidelines do not recommend electrolyte measurement either before or after bowel preparation for colonoscopy, it is important to consider that patients with known comorbidities and even those with unknown health conditions may be at high risk. Ugajin et al. reported a case of ventricular fibrillation during colonoscopy [18]. With the increasing number of colonoscopies for screening and follow-up, the prevalence of elderly patients—those with comorbidities and unknown health conditions—undergoing colonoscopies is expected to rise.

In daily practice, there are many different bowel preparation solutions. Some solutions can cause more significant electrolyte imbalances for high-risk patients. Saphira Z. et al. emphasized the importance of selecting the least harmful bowel preparation solution [50]. In our study, all participants used only one type of bowel preparation agent (X-M Diet solution) for bowel preparation. Because of this, we could not compare how different solutions affected ECG parameters. Colonoscopy under conscious sedation can diminish patient anxiety caused by the procedure. Midazolam and propofol, alone or combined with opioids, are commonly used [51,52]. When colonoscopy is performed with sedation, it should be kept in mind that anesthetic agents may also have arrhythmogenic effects [53].

Even though the patients included in our study were not specifically selected from high-risk patients with high arrhythmia potential, the study results were still statistically significant. In the literature, the limit value for a critical increase in QT value is stated as 500 ms [54]. In our study, although the mean QT and QTc values did not exceed 500 ms, there was a statistically significant increase in the values after colonoscopy compared to before colonoscopy. In patients who underwent colonoscopy, ambulatory rhythm recordings should be made after the procedure to search for new cut-off values of both QT and iCEB to provide a more accurate assessment for this population. Future studies focusing on high-risk patients may provide stronger evidence regarding the utility of iCEB in detecting arrhythmia risk.

### Study Limitations

This study’s primary limitation is that it was conducted in a single center with a small sample size. Because only one type of bowel preparation agent was used, the effect of different agents could not be compared. Additionally, patients were not monitored in the post-discharge period for procedure-related arrhythmias; 24 h Holter monitoring could have been more effective to uncover the silent cardiac arrhythmias related to colonoscopy.

## 5. Conclusions

Patients undergoing colonoscopy are often older, have more comorbid conditions, and are prescribed multiple medications. Data about complications such as electrolyte disturbances and cardiac arrhythmia related to bowel preparation and the colonoscopy procedure are limited and warrant further investigation. A quick and simple tool to assess arrhythmia risk is needed. ECG assessment of iCEB before discharge should be considered, especially for high-risk patients in routine care. Further multicenter studies with a large sample size including high-risk patients and long-term monitoring assessment are needed.

## Figures and Tables

**Table 1:** Baseline clinical characteristics of study population.

Variable		Descriptive Statistics
Age, years *		55.08 ± 11.23
Sex **	Male, n (%)	20 (55.56)
	Female, n (%)	16 (44.44)
Diabetes mellitus **		6 (16.67)
Hypertension **		9 (25.00)
Hyperlipidemia **		1 (2.78)
Coronary artery disease **		3 (8.33)
Heart failure **		0 (0.00)
Malignancy **		28 (77.78)
Urea *, mg/dL		29.68 ± 8.04
Creatinine ***, mg/dL		0.80 (0.50–1.20)
Glomerular filtration rate *, mL/dk/1.73 m^2^		94.62 ± 16.04
Sodium ***, mmol/L		139.50 (137–145)
Potassium *, mmol/L		4.49 ± 0.36
Calcium *, mg/dL		9.31 ± 0.48
Chloride *, mmol/L		105.68 ± 2.10
Glucose *, mg/dL		100.78 ± 15.74
White blood cell *, 10^9^/L		6.85 ± 2.09
Hemoglobin *, g/dL		13.37 ± 1.83
Platelets *, 10^9^/L		227.35 ± 69.49

Data given as the * mean ± standard deviation, ** n (%), or *** median (minimum value–maximum value).

**Table 2:** ECG parameters of the study population.

ECG Parameter	Pre-Colonoscopy	Post-Colonoscopy	*p*-Value
Heart rate, bpm **	74.5 (60–108)	68.5 (53–108)	0.021
QRS duration, ms **	91.0 (73–120)	90.5 (74–132)	0.317
P wave axis, degree *	57.2 ± 14.6	55.8 ± 14.1	0.598
QRS complex axis, degree *	32.3 ±34.9	19.4 ± 31.3	<0.001
T wave axis, degree **	43 (−67–124)	30.5 (−15–100)	0.021
PR interval, ms *	153.1 ±18.1	153.3 ± 18.3	0.919
QT interval, ms *	370.9 ± 27.8	398.7 ± 29.4	<0.001
QTc, interval, ms *	403.3 ± 24.2	427.4 ± 27.1	<0.001
iCEB *	4.1 ± 0.5	4.5 ± 0.6	<0.001
iCEBc *	4.4 ± 0.5	4.8 ± 0.7	<0.001

bpm: beats per minute; ms: milliseconds; QTc: corrected QT; iCEB: index of cardiac–electrophysiological balance; iCEBc: corrected index of cardiac–electrophysiological balance. Data given as the * mean ± standard deviationor ** median (minimum value–maximum value).

## Data Availability

All data are available without any restriction.
